# Mucoepidermoid Carcinoma in a Minor Salivary Gland in a Child

**DOI:** 10.1155/2013/615948

**Published:** 2013-07-21

**Authors:** Fatih Sengul, Sera Simsek, Binali Cakur

**Affiliations:** ^1^Department of Pedodontics, Faculty of Dentistry, Ataturk University, 25240 Erzurum, Turkey; ^2^Department of Oral, Dental And Maxillofacial Radiology, Faculty of Dentistry, Ataturk University, 25240 Erzurum, Turkey

## Abstract

Mucoepidermoid carcinoma (MEC), one of the most common salivary gland malignancies, is rare in children. MEC mainly occurs in the parotid gland, along with minor glands being the second common site, particularly in palate. Clinical, histological, and radiological findings of palatal MEC in a 12-year-old girl are presented with three-year follow-up. Pathologic lesions must be considered in differential diagnoses of intraoral asymptomatic lesions, and their detailed inspection should be taken into consideration.

## 1. Introduction

Mucoepidermoid carcinoma (MEC) is one of the most common salivary gland malignancies. As its name implies, MEC is composed of a mixture of cells, including mucus-producing, epidermoid or squamous, and intermediate types [1]. When MEC appears as asymptomatic swellings in minor salivary glands, being the second most common site of occurrence after the parotid gland, it can be located on palate, in retromolar area, floor of mouth, buccal mucosa, lips, and tongue [2–5].

Few series or case reports describing salivary gland tumors in the pediatric population have been published [6–8]. This report describes an additional case of a low-grade MEC affecting the palate of a 12-year-old girl.

## 2. Case Report

**Figure 1:**
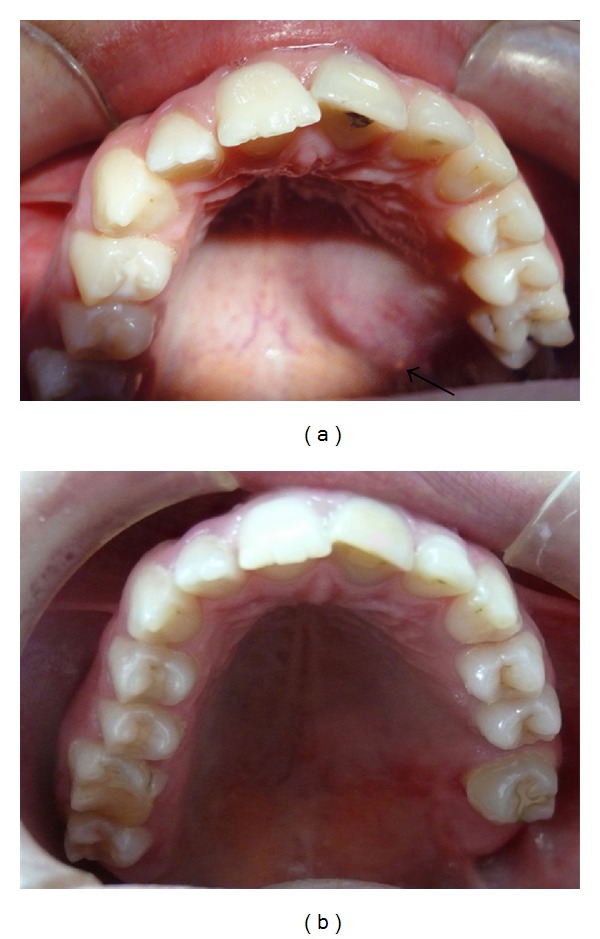
(a) Preoperative intraoral image of a mucoepidermoid carcinoma of the left posterior hard palate with 8 mm diameter and fistula opening (black arrow). (b) Postoperative (3 years) image of a reconstructed palate.

**Figure 2:**
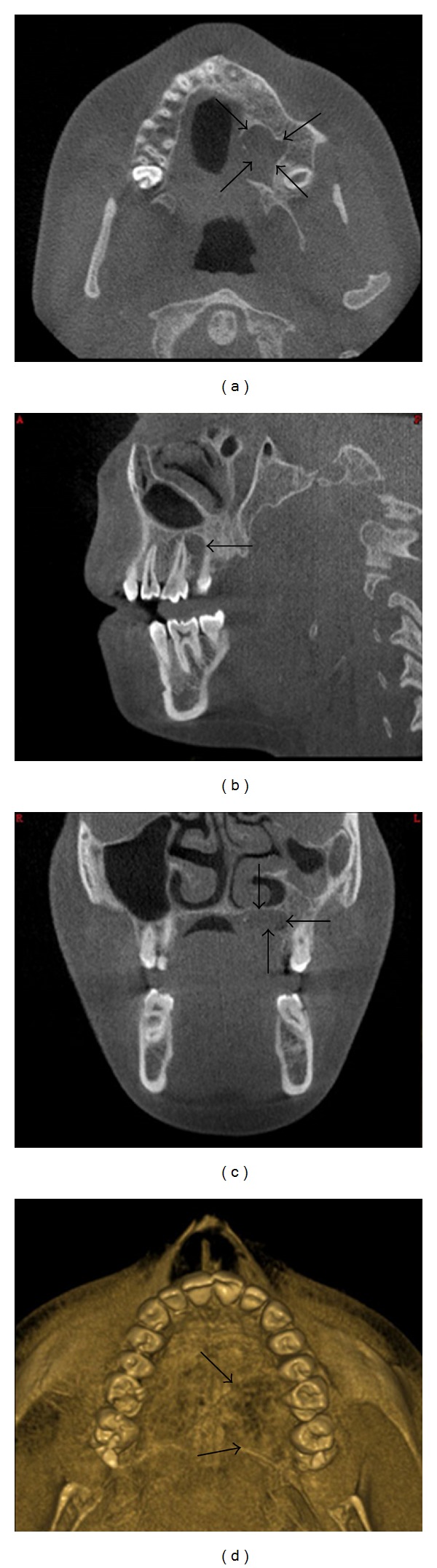
Dental volumetric tomography images of MEC (black arrows); (a) axial, (b) sagittal, (c) coronal, and (d) 3D image.

**Figure 3:**
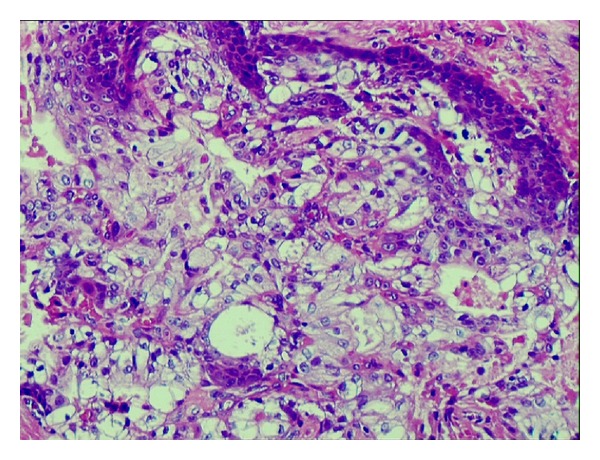
Histopathology showing mucous secreting cells and intermediate cells.

A 12-year-old girl patient visited the Department of Pediatric Dentistry, Ataturk University, Erzurum, Turkey, complaining of the pain in maxillar left central incisor (no. 11) and mandibular left lateral incisor (no. 22). She had a history of untreated Ellis II trauma and grade two mobility in these teeth for two years. Also, clinical examination revealed enlargement of the soft tissue in left posterior hard palate, with 8 mm diameter (Figure 1). During physical examination she had a firm, painless, and nontender mass, near the teeth 24 and 27, overlying the palate with normal color of mucosa, and the median palatal raphe was clearly identified. She denied any symptoms attributed to the mass. Adjacent teeth had no mobility or displacement, and electrical pulp test results were positive. In panoramic and periapical radiographs the alveolar bone had no resorption and the floor of the maxillary sinus appeared intact. Axial plain CT examination showed a moderately enhancing soft-tissue density lesion extending posteriorly and inferiorly destroying the hard palate and the alveolar process (Figure 2). No other abnormalities, including palpable submandibular and cervical lymph nodes, were found. Since fine needle aspiration yielded no fluid, a local oral surgeon performed an open biopsy and noticed a sinus opening near the foramen palatinum majus. Microscopic analysis revealed a low-grade mucoepidermoid carcinoma (Figure 3). By the end of the third year CT and oral examination showed no problem.

## 3. Discussion

Epithelial salivary gland neoplasms are rare both in adults and children, accounting for less than 3% of all head and neck tumors. 5% of these tumors occur in patients younger than 18 years old with girls mostly affected, while its occurrence in newborns is exceedingly rare [6, 9–11]. Malignancy seen in salivary gland tumors is 50% in children and 15–25% in adults [12].

As a typical intraoral presentation this malignancy has a painless and persistent enlargement, which lasts for about a year. Paresthesia, pain, and difficulty with swallowing are noted frequently when major salivary glands and tongue are involved. Intraoral lesions are observable as a localized fluctuant nodule with a bluish or reddish-purple, smooth, mucosal surface. Like in our study, mucus may be discharged from the tumor through a small sinus tract [8]. High-grade MEC epithelial cells are predominantly squamous whereas in low-grade, mucous cells predominate [13, 14]. Low-grade tumors are soft and compressible whereas, high-grade lesions may be quite firm and accompanied with ulceration, resorption of bone, and numbness of adjacent teeth [8]. 

Radiographically low-grade MECs are similar to benign mixed tumors. They demonstrate smooth margins and are characterized by cystic components containing mucin. On the other hand high-grade MECs have poorly defined margins, local infiltrations, and solid appearances [15].

The clinical and radiographic differential diagnosis of a palatal mass includes reactive and neoplastic lesions. In children, the most common of these entities is the palatal space abscess derived from pulpal necrosis. They are differentiated by tooth mobility, diffuse, erythematous swelling of sudden onset, suppuration, fluctuation in lesion size, and radiographic evidence of inflammatory pulpal disease. Mucocele is a frequently seen fluctuant reactive lesion of salivary glands, with transparent blue swelling including mucin. Deep mucoceles, often surrounded by a fibrous tissue wall, do not fluctuate, and if located at sites other than the lower lip cannot clinically and reliably be differentiated from salivary gland tumors. Our case was not similar to a mucocele because mucin discharged from a sinus opening. Palatal region vascular malformations like hemangiomas are differentiated by clinical examination and imaging. Neurofibroma and schwannoma are occasionally encountered as compressible or firm asymptomatic nodules and pink in color unless they are secondarily traumatized [8, 15].

 The tumor is dissected down to the periosteum to obtain adequate tumor-free margins [10]. But, if there is any evidence of bony involvement, removal of a portion of the jaw is necessary. Overall survival rate has been linked to histocytologic grade with 95%–100% in low-grade and 25%–43% in high-grade tumors [14]. Also, it should be considered that micromarsupialization, cryosurgery, and laser therapy are contraindicated in management of an intraoral submucosal mass/nodules in children particularly if the palate is involved [16–18]. These kinds of treatments may result in local spread of the tumor, and more aggressive surgery may be needed [8]. Based on low recurrence and mortality rates in surgical treatment of low-grade MEC, it seems that the treatment of our patient is adequate. 

Radiation therapy should be used judiciously in pediatric patients with high-grade histology, positive margins, and lymph node involvement, due to its long-term consequences as facial deformity, trismus, xerostomia, osteoradionecrosis, and risk of secondary malignancy. Chemotherapy was not used as an adjuvant therapy in our patient and does not currently have a role in the standard treatment of MEC patients [19].

There is a potential risk of the development of a mucoepidermoid carcinoma in parotid and minor salivary glands of children who have received chemotherapy and cranial irradiation [6, 20]. Moreover, survivors of the childhood cancer must be followed closely throughout their lifetime for the risk of developing a secondary malignancy following the treatment of childhood cancer [21].

## 4. Conclusions

In this case it is noteworthy that swellings in the palatal area which could resemble a dental abscess can cause unnecessary treatments, waste of time, and delay in diagnosis. Although mucoepidermoid carcinoma and other tumors in this region are exceedingly rare, patients with these kinds of swellings must be considered cautiously, and multidisciplinary approach can lead to successful treatment.
